# A retrospective cohort study on COVID-19 at 2 Los Angeles hospitals: Older age, low triage oxygenation, and chronic kidney disease among the top risk factors associated with in-hospital mortality

**DOI:** 10.1371/journal.pone.0268688

**Published:** 2022-06-22

**Authors:** Alisa Sato, Jeffrey Ludwig, Timothy Howell

**Affiliations:** 1 Department of Medicine, PIH Health Hospital Whittier & PIH Health Hospital Downey, Los Angeles, CA, United States of America; 2 Department of Mathematics, University of California Irvine, Irvine, CA, United States of America; 3 Department of Family Medicine, PIH Health Hospital Whittier & Clinical Informatics, PIH Health, Los Angeles, CA, United States of America; Osaka University Graduate School of Medicine, JAPAN

## Abstract

Los Angeles, California became a warzone of COVID-19 infections with up to one death every 10 minutes at the end of 2020. As resources thinned, and ICU beds and ventilators became scarce, physicians began agonizing over potentially rationing medical care. In this study, we conducted a retrospective cohort analysis of 7,429 confirmed COVID-19 positive patients from two community hospitals in Los Angeles, California between March 16, 2020 and June 9, 2021. We applied the Cox proportional hazards regression model to determine the risk factors most strongly associated with in-hospital mortality. Using the multivariable Cox proportional hazards model, there was a higher hazard ratio (HR) for mortality in patients who were older (age ≥60 years) [HR 2.189, 95% CI 1.991–2.407, p<0.001], had low triage oxygenation < 90% [HR 1.439, 95% CI 1.339–1.546, p<0.001], had chronic kidney disease (CKD) [HR 1.348, 95% CI 1.234–1.496, p = 0.001)], and who were obese (BMI ≥ 30 kg/m^2) [HR 1.221, 95% CI 1.155–1.340, p = 0.003)]. Overall, our study concluded that age ≥ 60 years, low triage oxygenation less than 90%, chronic kidney disease, and obesity were the top patient characteristics associated with increased mortality for both the univariate and multivariate Cox proportional hazards model analyses. Furthermore, by separating our data set into a development and validation set, we created a novel prediction tool to forecast in-hospital mortality and achieved 86% accuracy.

## Introduction

Late in December of 2019, there was a cluster of cases of pneumonia of unknown etiology possibly linked to the seafood and wet animal markets in Wuhan City, China [1, 2]. Soon thereafter, the severe acute respiratory syndrome coronavirus 2 (SARS-CoV-2) was identified as the cause of the highly contagious respiratory infection COVID-19 [3] which led to an explosive and deadly eruption of cases worldwide [4]. During the spring of 2020, in the United States of America (USA), New York City became an epicenter of COVID-19 infections [5]. As New York City struggled to deal with a massive shortage of beds, ventilators, oxygen, and protective equipment for healthcare workers, other states braced themselves for the upcoming onslaught of critically ill COVID-19 patients. California, with almost 40 million people, and the nation’s most populous state, seemed initially to be spared from the COVID-19 surge potentially from the stay-at-home and quarantine orders, masks, and closures of schools and non-essential businesses. Unfortunately, the 2020 holiday season brought about the inevitable: a tsunami of critically ill patients in respiratory failure bombarding hospitals throughout California.

As an Emergency Physician on the frontlines at two community hospitals in Los Angeles, California, PIH Health Whittier Hospital and PIH Health Downey Hospital, I experienced first-hand the wrath of COVID-19 as patients piled into our Emergency Departments at the start of the unprecedented surge in December 2020. Many hospitals in Los Angeles, California, America’s second-largest city [6] turned into warzones overnight. Although we had ample protective equipment and resources, many other rural areas did not. As resources spread thin in rural areas, ethical dilemmas arose about potentially rationing care to save those with the highest chances of survival.

With the sudden spike in COVID-19 cases, rapid triage evaluations became increasingly important. For example, what were the chances that an 81-year-old male with an oxygen saturation of 67% on room air with diabetes, chronic kidney disease, hypertension, and obesity would survive with aggressive mechanical intubation, high-flow oxygen, and admission? If we could quickly quantify his chances of survival based on a rapid 5-minute triage and found they were incredibly slim, would the family still want the patient to suffer through an invasive mechanical intubation, induced coma, and a high chance of dying alone in the hospital? Furthermore, could the 45-year-old female with hypertension and an oxygen saturation of 92% wait in the hallway with nasal cannula oxygen therapy until the more critical cases were stabilized? Under these circumstances, having a prediction tool to forecast the probability of death based on patient characteristics obtained during triage may have been useful to aid in medical decision-making, family discussions on expected prognosis, and intelligent allocation of scarce resources.

### Prior studies on risk factors associated with COVID-19 mortality

In the early stages of the COVID-19 pandemic, studies focused on determining what risk factors were associated with mortality. Increased age [3, 7–12], male sex [13] hypertension [7, 14, 15], diabetes, cardiovascular disease, chronic lung disease, cancer [3, 16–18], and frailty [19], were noted to be commonly cited risk factors associated with mortality for COVID-19 patients. In young patients, obesity was an additional risk factor leading to higher rates of complications, intubation, and death compared to non-obese patients [20, 21].

As more and larger scale datasets became available, CKD emerged as a profound, and one of the most significant risk factors associated with high mortality from COVID-19. The Global Burden of Disease collaboration, which collects health information worldwide, found that CKD was among the most prevalent risk factor for severe COVID-19 [22]. Clark [22] also found that in COVID-19 cases with advanced CKD, the normal risk factors for severe COVID-19 were less influential. Additionally, Williamson et al. [12] explained in a large-scale analysis of all the primary care electronic health records in England covering over 17.4 million patients, that age, male sex, cardiovascular disease, diabetes, respiratory disease, obesity, and cancer were among the risk factors for in-hospital death. However, CKD, including transplant and dialysis patients had the highest risk of mortality in this large-scale study. Jager et al. [23] found in their results from the ERA-EDTA (European Renal Association–European Dialysis and Transplant Association) registry that there was a very high mortality in dialysis and kidney transplant patients from COVID-19 infection as well. Hilbrands et al. [24] specifically found in the ERACODA (European Renal Association COVID-19 Database) that kidney transplant and dialysis patients with COVID-19 had a 21.3% and 25% probability of 28-day mortality respectively. Gibertoni et al. [25] found that the crude mortality rate among CKD patients with COVID-19 was 44.6%. Our study further investigated the effects of kidney disease on mortality for COVID-19 patients and additionally, we separated kidney disease into acute kidney disease and chronic kidney disease to see if there may be any differences in mortality for these subsets of patients.

Prehospital hypoxemia, or low oxygen levels, on room air obtained in triage in the Emergency Department or upon admission to the hospital has also been found to be an independent predictor of COVID-19 critical illness, ICU admission and in-hospital mortality [26–28]. As compared to traditional illnesses where hypoxemia correlates with significant dyspnea, or labored breathing, COVID-19-infected patients seem to have ‘silent hypoxia’ [29] where profoundly low oxygen levels are not commensurate with the patients’ relatively comfortable clinical appearance. However, despite the air hunger that COVID-19 patients may lack, the level of hypoxia measured in the pre-hospital setting and during triage evaluation has been previously shown to be a strong predictor of rapid decompensation and mortality [26, 30–33]. With this in mind, we investigated the effects of triage hypoxia (oxygen saturation < 90%) on our study population with the goal of using our results to improve accuracy for our in-hospital mortality prediction tool.

In this study, we performed a retrospective analysis of 7,429 confirmed COVID-19 patients between March 16, 2020, and June 9, 2021, at two community hospitals in Los Angeles, California to determine the risk factors most strongly associated with in-hospital mortality from COVID-19. We then separated our data set into a development and validation set to develop a clinical prediction tool to identify COVID-19 patients’ mortality risk based on rapid triage evaluation characteristics.

## Methods

### Study setting

This study included a multi-center retrospective review of charts extracted from the electronic medical records from both PIH Health Whittier and PIH Health Downey Hospitals. All patient data was fully anonymized prior to being accessed. This study had IRB exemption as it was a retrospective chart review and not a prospective clinical trial requiring patient enrollment. PIH Health Whittier Hospital is a 548-bed hospital, a comprehensive stroke center, and a ST elevation myocardial infarction (STEMI)-receiving center, while PIH Heath Downey Hospital is a 199-bed facility, a primary stroke center, and a STEMI-receiving center both serving patients in the Los Angeles, California area. Both hospitals utilize and share the same electronic medical health record, Allscrips Sunrise Clinical Manager, with similar hospital policies and protocols with most Emergency Physicians, Radiologists, Intensivists and some Hospitalists working at both facilities. Therefore, data from both hospitals were combined.

### Data collection

Information on the patients was extracted from the electronic medical record, including age, gender, body mass index (BMI), triage oxygen saturation (which was taken on room air), diabetes, hypertension, hyperlipidemia, acute and chronic kidney disease, chronic obstructive pulmonary disease (COPD), and asthma. These patient characteristics were determined a priori as some of the common risk factors associated with COVID-19 mortality from prior literature [7, 27, 34] and were easily obtained from the triage evaluation. The goal of relying on triage data was to implement a triage prediction tool to easily calculate the risk of in-hospital mortality upon patient arrival to the Emergency Department.

### SARS-CoV-2 testing and variants

The COVID-19 testing algorithm evolved during this retrospective data analysis given the novelty of the disease and the initial lack of testing supplies. During the early stages of the pandemic, starting March 16, 2020, our hospitals were only able to test symptomatic patients and many of these tests were send-out studies for COVID-19 after approval from the local Board of Health over the phone. As testing capabilities improved, we were able to test more patients using different testing modalities. By April 26, 2020, both of our hospitals started implementing in-hospital COVID-19 rapid PCR testing for some patients with the approval of our Infectious Disease or Pulmonology specialists. By May 14, 2020, all patients admitted to both facilities were required to have COVID-19 antigen or PCR testing initiated in the Emergency Department prior to moving to the inpatient beds. During the study period between March 16, 2020, and June 9, 2021, all patients with positive COVID-19 tests were included in our data set for analysis. Unfortunately, we were unable to distinguish between the different variants such as the alpha- or delta- variant with the testing capabilities we had available at our hospitals during the study period.

### Exclusion criteria

The initial dataset included a total of 10,741 confirmed COVID-19 positive patients on admission from both hospitals. A total of 3,312 patients had incomplete or missing data and a subset of patients who were transferred from the Emergency Department to other facilities such as pediatric patients, psychiatric patients or patients with HMO insurances requiring transfer to other hospitals were excluded from the study as we could not easily or reliably follow these patients’ clinical hospital course at other facilities. Further exclusion criteria included: patients who did not have a full data set of information (e.g., missing data points for the risk factors), patients who had an obvious mistake in data entry outside of normal ranges, or patients with duplicate charts, and these were removed from the data set. We accepted that this data was incomplete after the data was extracted from the computer system and thoroughly reviewed. The remainder included a total of 7,429 patients in the sample study. Providers administering treatments during the hospital course were neither blinded nor controlled, as this was a retrospective chart review.

### Defining study outcomes

The primary outcome was a binary result: the patient either survived or died during hospitalization. COVID-19 patients were classified as survivors if they were either discharged home or to an appropriate rehabilitation or nursing facility. Patients were classified as non-survivors if they died during their hospitalization. We did not follow the clinical course of the patients after they were discharged from the hospital.

### Defining kidney disease in this study

Classically, patients with kidney disease are most easily identified as those with impaired renal function with a decreased glomerular filtration rate (GFR) of less than 60 mL/min/1.73m^2 [35]. Rather than combining all patients with ‘kidney disease’ with a decreased glomerular filtration rate, our patient population was separated into those with acute kidney disease and chronic kidney disease.

New impairments of renal function, or acute kidney disease, found on admission were described as any patient with a GFR of less than 60 mL/min/1.73m^2 (with no prior GFR on file to compare to), >25% decrease in GFR compared to prior results, or an increase in serum creatinine ≥ 1.5-fold from baseline with no prior documentation or history of kidney disease. During the data collection process, these patients were identified based on chart review with discharge diagnoses or summaries including acute kidney disease and appropriate synonyms.

Chronic kidney disease patients were identified as patients with a prior history of chronic kidney disease previously documented in the chart or described by the patient on intake triage questioning. Typically, the criteria for a diagnosis of chronic kidney disease includes either a decreased GFR of less than 60 mL/min/1.73m^2, or markers of kidney damage including albuminuria, urine sediment abnormalities, electrolyte disturbances due to tubular disorders, histologic and structural abnormalities detected by imaging present for greater than 3 months with health implications [36]. Historically, there are stages of chronic kidney disease to classify the severity: stage 1 (GFR 90 mL/min/1.73m^2 or higher), stage 2 (GFR 60–89 mL/min/1.73m^2), stage 3a (GFR 45–59 mL/min/1.73m^2), stage 3b (GFR 30–44 mL/min/1.73m^2), stage 4 (GFR 15–29 mL/min/1.73m^2), and stage 5 (GFR less than 15 mL/min/1.73m^2), with stage 5 including end stage renal disease and those requiring renal replacement therapies such as dialysis. We included all these patients in the chronic kidney disease group for data analysis and did not separate these patients based on their stage of chronic kidney disease. Patients with chronic kidney disease usually presented to the hospital with a prior diagnosis by a primary doctor or nephrologist in the outpatient setting, especially since this requires a minimum of 3 months of data to diagnose. During data collection, we identified these patients by thorough chart review based on their discharge diagnoses and past medical history.

### Statistical analysis

Continuous variables included age, BMI and triage oxygenation. All these continuous variables were divided into 2 categories with a defined threshold. Age was grouped into binary categories: age ≥60 years and age <60 years. BMI was grouped into binary categories as well: BMI ≥30 kg/m^2 was used to define obese patients, and BMI <30 kg/m^2 was used to define non-obese patients. Triage oxygen levels were also categorized into two groups: triage O2 saturation <90% was used to define the moderate to severely hypoxic group and triage O2 saturation ≥ 90% was used to define the mildly hypoxic group. All other categorical variables (gender, diabetes, hypertension, hyperlipidemia, acute kidney disease, chronic kidney disease, chronic obstructive pulmonary disease, asthma) were reported as a number (0 or 1) for statistical analysis.

### Prediction tool

One of the goals of this study was to examine data from 7,429 patients with COVID-19 and identify risk factors from the triage examination that correlated with poor outcomes, specifically in-hospital mortality. We did not include risk factors that would need additional time to obtain such as laboratory values, imaging studies, urinalysis tests, electrocardiograms, treatment modalities, etc. with the intent of creating a tool for emergency medicine practitioners to rapidly predict patient outcomes after a 5-minute triage on initial presentation. To develop the prediction tool, we utilized the same dataset (7,429 total patients) and divided the dataset into a development group and a validation group. Among the total 7,429 patients, 6,000 (80.76%) patients were randomly assigned to the development group and the remaining 1,429 (19.24%) patients were assigned to the validation group. Logistic regression was used on the development group to compute coefficients for the prediction model. If our estimate of the probability of in-hospital death was ≥ 47%, we predicted death. If our estimate of the probability of in-hospital death was <47%, we predicted survival. Using this threshold methodology, we achieved 86% accuracy when testing this prediction model on our validation group.

## Results

### Study patients and demographics

Of the total 7,429 patients (Table 1), the median age was 58 years old with the youngest <1 year old and oldest 103 years old. The mean age was 56.4 years old and the sample standard deviation of the age distribution was 19.23. Older patients (age ≥60 years) included 3,508 (47.2%) of the study and younger patients (age <60 years) included 3,921 (52.8%) of the total study population. 18.6% (652 out of 3,508 patients) of the older cohort of patients age ≥ 60 years died in the hospital compared to only 3.9% (153 out of 3,921 patients) of the younger cohort of patients age <60 years.

**Table 1:** Descriptive characteristics of study patients (n = 7,429), demographics, and survival status.

Variable	Total, *n* (%)	Alive, *n* (%)	Died, *n* (%)
All Study Patients	7,429 (100%)	6,624 (89.2%)	805 (10.8%)
Age			
Age <60 years	3,921 (52.8%)	3,768 (96.1%)	153 (3.9%)
Age ≥60 years	3,508 (47.2%)	2,856 (81.4%)	652 (18.6%)
Gender			
Female	3,573 (48.1%)	3,251 (91.0%)	322 (9.01%)
Male	3,856 (51.9%)	3,374 (87.5%)	482 (12.5%)
BMI			
BMI ≥30 kg/m^2	3,510 (47.2%)	3,152 (89.8%)	358 (10.2%)
BMI<30 kg/m^2	3,919 (52.8%)	3,472 (88.6%)	447 (11.4%)
Triage Oxygenation			
Triage O2 ≥ 90%	5,948 (80.1%)	5,571 (93.7%)	377 (6.34%)
Triage O2 < 90%	1,481 (19.9%)	1,053 (71.1%)	428 (28.9%)
Health Conditions			
Diabetes	2,653 (35.7%)	2,204 (83.1%)	448 (16.9%)
Hypertension	2,502 (33.7%)	2,189 (87.5%)	312 (12.5%)
Hyperlipidemia	1,534 (20.6%)	1,073 (78.7%)	460 (12.3%)
Acute Kidney Disease	70 (0.01%)	43 (61.4%)	27 (38.6%)
Chronic Kidney Disease	840 (11.3%)	620 (73.8%)	220 (26.2%)
Chronic Obstructive Pulmonary Disease	229 (3.1%)	178 (77.7%)	51 (22.3%)
Asthma	439 (5.9%)	402 (91.6%)	37 (8.4%)

There were slightly more male (3,856, 51.9%) patients compared to female (3,573, 48.1%) patients in this study. Males had worse outcomes with a 12.5% (482 out of 3,856 patients) death rate compared to females with a 9.01% (322 out of 3,573 patients) death rate.

Regarding triage oxygen levels, 5,948 (80.1%) of the patients had oxygen levels ≥ 90%, and 1,481(19.9%) of the patients presented with oxygen levels <90%. For the moderate to severely hypoxic patients (oxygen levels <90%), 28.9% (428 out of 1481 patients) of them died in the hospital. This is in stark contrast to the patients who presented with higher triage oxygen levels ≥ 90% where only 6.34% (377 out of 5,948 patients) of them died.

The most common comorbidity was obesity with 3,510 (47.2%) of the patients having a BMI ≥30 kg/m^2. The median BMI was 29.5 kg/m^2 (min 9.4 kg/m^2, max BMI 99.3 kg/m^2). The next common comorbidity was diabetes mellitus with 2,653 (35.7%) of the patients, followed by hypertension with 2,502 (33.7%) of the patients, hyperlipidemia with 1,534 (20.6%) of the patients, and acute and chronic kidney disease with a total of 910 (12.2%) of the patients, asthma with 439 (5.9%) of the patients, and COPD with 229 (3.1%) of the patients. The median triage oxygen saturation was 95% (min of 25%, max of 100%). Table 1 summarizes the patient demographics and in-hospital mortality rates for this study.

### Patient enrollment

**Fig 1:**
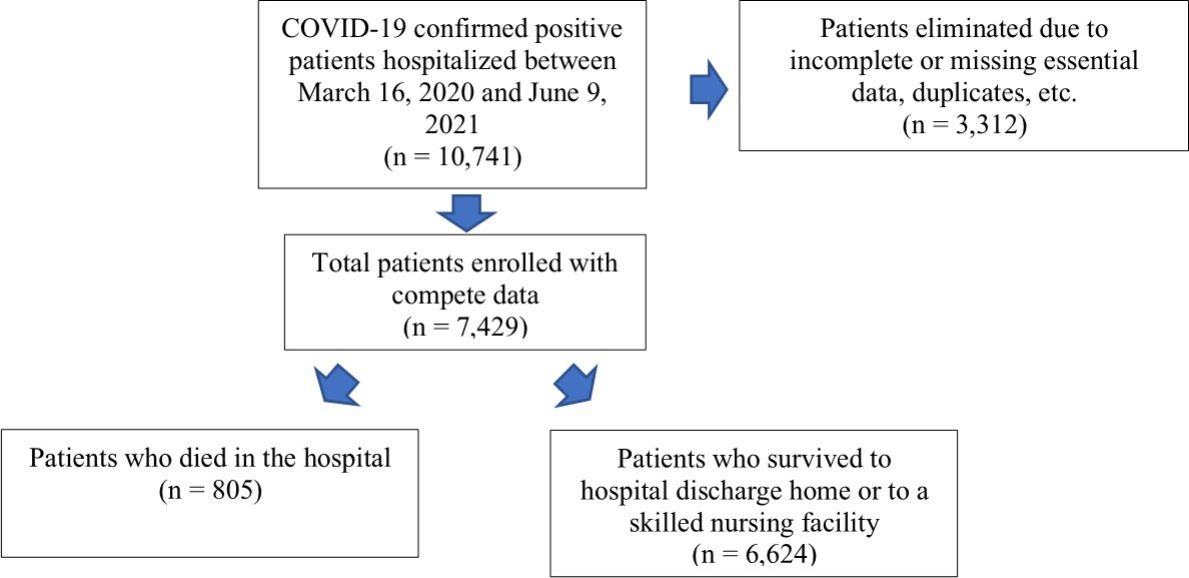
Patient enrollment and overall outcome.

Fig 1 shows the flowchart of patient enrollment, including 7,429 patients with complete datasets and those who were excluded (n = 3,312). 805 (10.8%) patients died during their hospital stay and 6,624 (89.2%) patients survived till discharge.

### Cox proportional hazards regression analysis

Both univariate and multivariate Cox proportional hazards models were used to determine the risk factors for mortality and were presented as hazard ratios (HR) with 95% CIs. Of the statistically significant results, with a p-value <0.05, the univariate analysis showed that age ≥ 60 years [HR 4.470, 95% CI 4.085–4.891, p<0.001], chronic kidney disease [HR 1.560, 95% CI 1.442–1.690, p<0.001], triage oxygenation <90% [HR 1.397, 95% CI 1.301–1.501, p<0.001], diabetes mellitus [HR 1.173, 95% CI 1.092–1.259, p = 0.025], and hyperlipidemia [HR 1.172, 95% CI 1.090–1.259, p = 0.028] had higher hazards of mortality while being female and hypertension had lower hazards of mortality.

In the multivariable Cox proportional hazards model (Table 2), older patients with age ≥ 60 years [HR 2.189, 95% CI 1.991–2.407, p<0.001], patients with triage oxygenation <90% [HR 1.439, 95% CI 1.339–1.546, p<0.001], patients with chronic kidney disease [HR 1.348, 95% CI 1.234–1.496, p = 0.001] and patients with a BMI ≥30 kg/m^2 [HR 1.221, 95% CI 1.155–1.340, p = 0.003] had higher hazards of mortality. On the other hand, hypertension was associated with a lower hazard ratio.

**Table 2:** Univariable and multivariable Cox proportional hazards model for in-hospital mortality among COVID-19 patients.

	Univariable HR	95% CI	p-value		Multivariable HR	95% CI	p-value
Age <60 yrs	1 (Ref Group)			Age <60 yrs	1 (Ref Group)		
Age ≥60 yrs	4.470	4.085–4.891	<0.001	Age ≥60 yrs	2.189	1.991–2.407	<0.001
Female	1 (Ref Group)			Female	1 (Ref Group)		
Male	0.874	0.813–0.939	0.348	Male	1.041	0.971–1.126	0.549
BMI < 30 kg/m^2	1 (Ref Group)			BMI < 30 kg/m^2	1 (Ref Group)		
BMI ≥30 kg/m^2	1.083	1.009–1.163	0.262	BMI ≥30 kg/m^2	1.221	1.155–1.340	0.003
Triage O2 ≥ 90%	1 (Ref Group)			Triage O2 ≥ 90%	1 (Ref Group)		
Triage O2 < 90%	1.397	1.301–1.501	<0.001	Triage O2 < 90%	1.439	1.339–1.546	<0.001
Diabetes	1.173	1.092–1.259	0.025	Diabetes	1.043	0.971–1.129	0.544
Hypertension	0.846	0.786–0.909	0.020	Hypertension	0.846	0.769–0.911	0.036
Hyperlipidemia	1.172	1.090–1.259	0.028	Hyperlipidemia	1.031	0.955–1.110	0.696
Acute Kidney Disease	1.285	1.056–1.564	0.201	Acute Kidney Disease	0.890	0.724–1.094	0.573
Chronic Kidney Disease	1.560	1.442–1.690	<0.001	Chronic Kidney Disease	1.348	1.234–1.496	0.001
Chronic Obstructive Pulmonary Disease	1.316	1.139–1.521	0.058	Chronic Obstructive Pulmonary Disease	1.16	1.023–1.371	0.249
Asthma	0.832	0.703–0.985	0.276	Asthma	0.930	0.784–1.103	0.669

### Prediction tool: Dividing the dataset into development and validation sets

Using the same dataset of a total of 7,429 patients, we divided the dataset into a development set (Table 3) of 6,000 randomly selected patients and a validation set (Table 4) of 1,429 patients.

**Table 3:** Descriptive characteristics of study patients, demographics, and survival status in the prediction tool development (n = 6,000 patients) dataset.

Variable	Total, *n* (%)	Alive, *n* (%)	Died, *n* (%)
All Study Patients	6000 (100%)	5352 (89.2%)	648 (10.8%)
Age			
Age <60 years	3304 (55.1%)	3175 (96%)	129 (4%)
Age ≥60 years	2696 (44.9%)	2177 (80.7%)	519 (19.3%)
Gender			
Female	2853 (47.5%)	2605 (91.3%)	248 (8.7%)
Male	3147 (52.5%)	2747 (87.3%)	400 (12.7%)
BMI			
BMI ≥30 kg/m^2	2844 (47.4%)	2558 (89.9%)	286 (10.1%)
BMI<30 kg/m^2	3156 (52.6%)	2794 (88.5%)	362 (11.5%)
Triage Oxygenation			
Triage O2 ≥ 90%	4847 (80.8%)	4,540 (93.7%)	307 (6.3%)
Triage O2 < 90%	1153 (19.2%)	812 (70.4%)	341 (29.6%)
Health Conditions			
Diabetes	2091 (34.8%)	1728 (82.6%)	363 (17.4%)
Hypertension	1967 (32.8%)	1714 (87.1%)	253 (12.9%)
Hyperlipidemia	1159 (19.3%)	899 (77.6%)	260 (22.4%)
Acute Kidney Disease	58 (0.01%)	35 (60.3%)	23 (39.7%)
Chronic Kidney Disease	649 (10.8%)	472 (72.7%)	177 (27.2%)
Chronic Obstructive Pulmonary Disease	179 (3%)	138 (77.1%)	41 (22.9%)
Asthma	352 (5.9%)	327 (92.9%)	25 (7.1%)

**Table 4:** Descriptive characteristics of study patients, demographics, and survival status in the prediction tool validation (n = 1,429 patients) dataset.

Variable	Total, *n* (%)	Alive, *n* (%)	Died, *n* (%)
All Study Patients	1,429 (100%)	1,272 (89.0%)	157 (11.0%)
Age			
Age <60 yrs	617 (43.2%)	592 (95.9%)	25 (4.1%)
Age ≥60 yrs	812 (56.8%)	680 (83.7%)	132 (16.3%)
Gender			
Female	720 (50.4%)	646 (89.7%)	74 (10.3%)
Male	709 (49.6%)	626 (88.3%)	83 (11.7%)
BMI			
BMI ≥30 kg/m^2	666 (46.6%)	595 (89.3%)	71 (10.7%)
BMI<30 kg/m^2	763 (53.4%)	677 (88.7%)	86 (11.3%)
Triage Oxygenation			
Triage O2 ≥ 90%	1,101 (77.0%)	1,031 (93.6%)	70 (6.4%)
Triage O2 < 90%	328 (22.9%)	241 (73.5%)	87 (26.5%)
Health Conditions			
Diabetes	562 (39.3%)	476 (84.7%)	86 (15.3%)
Hypertension	535 (37.4%)	476 (89%)	59 (11%)
Hyperlipidemia	375 (26.2%)	309 (82.4%)	66 (17.6%)
Acute Kidney Disease	12 (0.008%)	8 (66.7%)	4 (33.3%)
Chronic Kidney Disease	191 (13.4%)	148 (77.5%)	43 (22.5%)
Chronic Obstructive Pulmonary Disease	50 (3.5%)	40 (80%)	10 (20%)
Asthma	86 (6%)	74 (86%)	12 (14%)

The data from the development group of 6,000 patients was used to train the prediction model using logistic regression [37] (Table 5). Once the coefficients of the prediction model were computed, we were able to compute an estimate of the probability of in-hospital death based on triage information from these 10 explanatory variables (age≥60 years, gender, BMI≥ 30 kg/m^2, triage oxygenation <90%, diabetes, hypertension, hyperlipidemia, chronic kidney disease, COPD, and asthma).

**Table 5:** Prediction tool: List of explanatory variables and coefficients computed using the development set (n = 6,000 patients).

Explanatory Variables	Coefficients
Age ≥ 60 years	1.463
Gender	0.494
BMI ≥ 30 kg/m^2	0.249
Triage oxygenation <90%	2.255
Diabetes	0.195
Hypertension	0.033
Hyperlipidemia	0.504
Chronic Kidney Disease	0.870
Chronic Obstructive Pulmonary Disease	0.333
Asthma	-0.090

Each of these explanatory variables contributed to the probability of death based on the logistic regression prediction model. The outcome was binary, meaning the model could either predict death or survival. Therefore, we applied a threshold of 47% to the probability of death: if the estimated probability of death was ≥47%, we predicted death, and conversely, if the estimated probability of death was <47%, we predicted survival.

We then used the 1,429 patients in the internal validation group to test the model. Using the coefficients obtained through logistic regression on the development group of 6,000 patients, the prediction model accurately predicted death in 86% of the 1,429 patients in the validation group.

One of the merits of this prediction model is that it can be computed simply using information obtained in triage. Conceivably, one could input this data into a phone application to allow practitioners to rapidly calculate the probability of death and aid in triage evaluations.

## Discussion

### Age

Older age was identified early in the pandemic as a significant risk factor for mortality. In one of the earliest, large-scale reports on the COVID-19 outbreak in China published in JAMA [3] on February 24, 2020, Wu et al. describes the findings from the Chinese Center for Disease Control and Prevention. Based on 72,314 case records and 44,672 confirmed cases of COVID-19, this study found a case-fatality rate (CFR) of 2.3% overall (1,023 of 44,672 confirmed cases). For the older patients aged 70–79 years, the CFR was higher at 8.0% and for patients age ≥ 80 years, the CFR was disproportionately high at 14.8%. As the pandemic spread to Italy, COVID-19 seemed to be more lethal, but may have been skewed by the older average population in Italy compared to China. In a study by Onder et al. [11], the authors revealed the overall CFR in Italy (7.2%) to be more than 3 times the CFR in China (2.3%). Furthermore, the CFR for patients in Italy age 70–79 years was 12.8% compared to 8.0% in the similar aged cohort of patients in China. Given that about 23% of the Italian population was aged 65 and older at the time of the pandemic, it was thought that older age had a significant role in explaining the higher lethal rate of COVID-19 in Italy compared to China [11]. Additionally, in a large-scale study in England by Williamson et al. [12] examining COVID-19 deaths in 10,926 patients, the authors found that increasing age was strongly associated with increased risk of death with those aged > 80 years old having a more than 20-fold increased risk of mortality compared to those aged 50–59 years old. Richardson et al. [38] also demonstrated early in the pandemic in a case series of 5,700 patients with COVID-19 in New York that the mortality rates were much higher for those older than 65 years and especially for those who received mechanical ventilation [38].

In comparison to these large-scale international studies, our study corroborated with these results that older age had a strong association with mortality. In our study population of 7,429 total COVID-19 positive patients who presented to our hospitals, 3,508 (47.2%) of our patients were age ≥ 60 years compared to 3,921 (52.8%) of our patients were age <60 years. 18.6% (652) of our older population age ≥ 60 years compared to 3.9% (153) of our younger population age <60 years died in the hospital. We can’t directly compare these fatality rates to the previously mentioned international studies [3, 11, 12] as our population only included sicker patients requiring hospitalization with COVID-19 and did not include patients who were well appearing, without significant hypoxia, and who did not feel sick enough to present to the Emergency Department. Based on our Cox proportional hazards model, we found that older age classified as age ≥ 60 years was the single most influential factor for in-hospital mortality for both the univariate [HR 4.470, 95% CI 4.085–4.891, p<0.001] and multivariate analyses [HR 2.189, 95% CI 1.991–2.407, p<0.001]. The results of our study are consistent with the findings in many prior studies, further solidifying the argument that older age has a profound impact on mortality for COVID-19 patients [3, 7–12].

There are many possible explanations for the higher mortality rate in older patients with COVID-19 infections. Studies have suggested that the aging process leads to a host of changes in the immune system. Immunosenescense, or a gradual decline in immune function, and inflammaging, or chronic increase in systemic inflammation leads directly to a decline in protective responses to infections [39, 40]. Additionally, the increased rate of comorbidities in older patient populations further compromise the immune system and the ability to recognize, fight, and clear infections. It has also been proposed that older patients have a propensity for pro-inflammatory cytokine production and may lead to an exaggerated innate immune response with worsened COVID-19 outcomes [41].

### Triage oxygenation

Unlike many other respiratory diseases, COVID-19 ultimately created a new phenomenon coined ‘silent hypoxia’ where profoundly low oxygenation levels were not accompanied with the anticipated level of respiratory distress. We now know, however, that initial low oxygen levels on presentation or on admission to the hospital are often highly correlated with poor outcomes [31, 32]. In this study we used the cutoff of 90% oxygen saturation to identify those with mild hypoxia (triage oxygenation ≥ 90%) and those with moderate to severe hypoxia (triage oxygenation <90%). One of the goals of the study was to use the triage oxygenation to highlight a subset of patients who were easily identified as patients who would likely decompensate and do poorly during admission. Using the triage oxygenation as a continuous variable would make this distinction between patients less noticeable and therefore less useful as a prediction measure.

In our study, we identified 5,948 patients (80.1%) with a triage oxygenation ≥ 90% and 1,481 patients (19.9%) with a triage oxygenation <90%. Only 6.34% (377 out of 5948) of the patients with mild hypoxia died, compared to 28.9% (428 out of 1481) of the patients with moderate to severe hypoxia died during hospitalization. Therefore, the patients presenting with moderate to severe hypoxia with a triage oxygenation <90% had a 4.5-times higher (28.9% vs. 6.34%) incidence of in-hospital mortality as compared with the patients with mild hypoxia with a triage oxygenation ≥ 90%.

Using the Cox proportional hazards model, we found with our univariable analysis that the hazards ratio for moderate to severe hypoxia was 1.397 (95% CI 1.301–1.501, p<0.001) and with our multivariable analysis, that the hazards ratio was 1.439 (95% CI 1.339–1.546, p<0.001) both of which were clinically significant. Our study confirms that triage oxygen levels less than 90% correlate with poor outcomes and increased mortality. This may encourage emergency medical professionals and triage nurses to be vigilant in obtaining accurate intake oxygen levels and triage those with low oxygen less than 90% to the highest level of care for aggressive treatment in the Emergency Department understanding that they have a 28.9% chance of death. Consequently, patients with triage oxygen levels ≥ 90% in our study only had a 6.34% rate of mortality and therefore may be placed on low levels of oxygen therapy (e.g., nasal cannula 1–4 liters/min of oxygen) and can potentially wait to be evaluated during times of a surge and lack of personnel, treatment modalities, or space.

### Kidney disease

Many prior studies have demonstrated that chronic kidney disease is one of the risk factors associated with COVID-19 mortality [12, 22–25]. In the large-scale study by Williamson et al. [12] evaluating 1,007,383 COVID-19 patients in England with reduced kidney function with a GFR 30–60 mL/min/1.73m^2, and 78,093 COVID-19 patients with GFR <30 mL/min/1.73m^2, there was a HR of 1.33 (95% CI 1.28–1.4) and HR 2.52 (95% CI 2.33–2.72) respectively. Furthermore, in a large-scale study in the United States of America with 31,461 COVID-19 patients, Harrison et al. [42] showed that renal disease [OR 2.13, 95% CI 1.84–2.46, p<0.001] was the third most significant risk factor associated with mortality behind native Hawaiian/Pacific Islander ethnicity [OR 3.63, 95% CI 1.75–7.52, p = 0.001] and moderate/severe liver disease [OR 2.62, 95% CI 1.53–4.47, p<0.001] in their multivariate analysis. Also, in a study by Petrilli et al. [33] based out of New York with a cohort of 5,279 COVID-19 patients, chronic kidney disease, along with older age, male sex, heart failure and obesity were associated with hospital admission and risk of progression to critical illness. Although these and many other studies confirm the significant impact of chronic kidney disease on COVID-19 mortality, there has been less focus on delineating between acute and chronic kidney disease and their relative impacts on COVID-19 mortality.

In our study, we separated patients with kidney disease into two categories: acute and chronic kidney disease to determine if there were any differences in outcome. Overall, there were 840 (11.3%) total patients with chronic kidney disease of which 220 (26.2%) died and there were only 70 (0.01%) total patients with acute kidney disease of which 27 (38.6%) died. Using univariable Cox proportional hazards analysis, chronic kidney disease had a hazards ratio of 1.560 (95% CI 1.442–1.690, p<0.001) which was clinically significant, but acute kidney disease had a hazards ratio of 1.285 (95% CI 1.056–1.564, p = 0.201) which was not clinically significant. Using multivariable Cox proportional hazards analysis, chronic kidney disease had a hazards ratio of 1.348 (95% CI 1.234–1.496, p = 0.001) which was clinically significant, but again, acute kidney disease had a hazards ratio of 0.89 (95% CI 0.724–1.094, p = 0.573) which was not clinically significant. In summary, after dividing the kidney disease patients into sub-groups of acute kidney disease and chronic kidney disease, we found that chronic kidney disease was associated with a higher hazards ratios and mortality rates, but acute kidney disease analysis failed to achieve clinical significance possibly due to the low number of patients identified with acute kidney injury in this study. Both subgroups, however, still had a significantly high death rate of 38.6% and 26.2% for acute and chronic kidney disease respectively. Our study still corroborates with prior studies that renal impairment, and specifically chronic kidney disease, is a major risk factor for COVID-19 mortality [12, 24].

If patients with chronic kidney disease have such a higher rate of mortality with COVID-19, it would be highly recommended for patients to obtain baseline renal function tests to help identify this often silent risk factor. Most people with mild chronic kidney disease will unfortunately not have any obvious signs or symptoms of this disease. The Center for Disease Control (CDC) estimates that approximately 37 million people in the USA have chronic kidney disease and about 40% of people with severely reduced kidney function are unaware of having chronic kidney disease [43]. On a global scale, Jager et al. [23] estimates that those with chronic kidney disease, acute kidney injury and those on renal replacement therapy exceeds 850 million which is twice the number of people estimated worldwide to have diabetes.

Unfortunately, studies have shown that patients with chronic kidney disease are at an increased risk for pneumonia as compared to the general population [44] and are at increased risk for severe infection from COVID-19 [45, 46]. One study by Perico et al. [47] suggests with severe COVID-19 infection, there may be an unbalanced innate immune response in which the endothelial dysfunction, coagulopathy and thrombosis leads to microangiopathy that is particularly harmful to the kidney and contributes to the cascade of multi-organ failure and death. Given this preponderance of evidence that underlying chronic kidney disease may lead to a much higher mortality rate for COVID-19 patients we suggest that patients seek baseline laboratory studies to help identify chronic kidney disease and work with a primary care providers to modify factors and help mitigate progression of this disease.

### BMI

In our cohort, we defined obesity as a BMI ≥ 30 kg/m^2. There were 3,510 (47.2%) patients with a BMI ≥ 30 kg/m^2, comprising almost half of the admitted COVID-19 positive patients. Prior studies have shown that obese patients have a profound increased risk of mortality from COVID-19 [48–51] especially for the morbidly obese patients defined as BMI > 40kg/m^2 [52]. One study by Popkin et al. [53] involved a meta-analysis of 75 studies on the relationship with obesity and COVID-19 with the risk of mortality. Popkin et al. [53] found through pooled analysis that individuals with obesity and COVID-19 were at a higher risk for hospitalization, ICU admission, and for mortality with a 48% increase in deaths (OR 1.48, 95% CI 1.22–1.8, p<0.001). In a report by the Center for Disease Control and Prevention published on March 12, 2021 [54], it was reported that among 148,494 U.S. adults with COVID-19, the severity of disease increased with a higher BMI, and obesity alone was a risk factor for hospitalization and death, especially in those aged <65 years old.

Of the 3,510 (47.2%) patients in our study with obesity, 358 (10.2%) died in the hospital. In our multivariable Cox proportional hazards model, obesity had a HR of 1.221 [95% CI 1.155–1.340, p = 0.003] and thus had a significant association with in-hospital mortality. However, in our univariable Cox proportional hazards model, our hazards ratio for obese patients was 1.083 (95% CI 1.009–1.163, p = 0.262) and did not reach clinical significance. Although obesity was a risk factor increasing the risk of in-hospital mortality, it was not as profound of a risk factor as older age ≥ 60, triage oxygenation <90%, and chronic kidney disease in our current study.

### Prediction tool

Our prediction tool accurately predicted death in 86% of the 1,429 patients. The goal of this prediction tool was to utilize data from the medical screening examination in triage to generate a probability of death when the patient is first seen in the Emergency Department. We were careful to select parameters that were easily obtained during the triage evaluation to simplify the process. This prediction tool could provide useful information on the probability of death immediately after triage to aid in family discussions, allocating appropriate resources, and potentially rationing care during times of a surge. Because this is an internal validation tool, it would be useful for the patients specifically admitted to PIH Health Whittier and PIH Health Downey Hospitals with COVID-19 to predict in-hospital mortality of new patients during a similar time period and with similar COVID-19 variant infections. Future models of this sort may consider expanding parameters to include more triage risk factors and a larger subset of patients to potentially improve accuracy. External validation using data from other hospitals may also expand the use of this prediction tool.

### Limitations

Our retrospective study collected data on a novel viral disease during a prolonged time period between March 16, 2020, and June 9, 2021. In the first few months of our study, COVID-19 testing was not readily available and required approval from the local Board of Health and/or the Infectious Disease specialists with a long turnaround time. We may not have captured all the COVID-19 patients during this time given the lack of testing supplies. As the pandemic continued, new variants appeared, and there was a transition between the alpha- and delta- COVID-19 variants, yet we were unable to test specifically for each variant at our hospitals. Variants may have played a significant role in the patient outcome of in-hospital mortality, and we were unable to account for this bias in our data set.

In-hospital treatments were also highly variable and constantly evolving. Patients who were hospitalized with COVID-19 in the summer of 2021 were treated with newly developed medications and techniques, which were not available early in the pandemic. Additionally, vaccinations became available in 2021, which likely affected the hospital course of patients with breakthrough infections who were hospitalized. Therefore, the subset of the patients hospitalized in the later stages of the pandemic may have a biased clinical course with potentially better chances of survival.

Additionally, our initial cohort of 10,741 COVID-19 patients had missing data and therefore could not be included in our study. We excluded 3,312 patients, many of whom did not have their height entered in the electronic medical record to calculate their BMI. Although this was a large percentage of our total cohort, we still achieved statistical significance in many of our Cox proportional hazards models given the large number of patients with complete data. We considered including race and socioeconomic status, as these factors have been shown to have an effect on mortality, however a large proportion of the computer data lacked this specific information and would have severely limited our statistical analysis. Future studies may focus on specifically collecting this information a priori in a prospective trial.

Lastly, our study evaluated in-hospital mortality but did not follow patients after they were discharged home. There may have been a significant number of patients who survived till discharge but died at home and were counted as “survived”. Future directions include conducting a prospective clinical trial enrolling patients from the start of their triage in the Emergency Department to obtain complete and accurate risk factor data and following these patients long-term after their hospitalization. Improvements in prediction tools using triage characteristics, machine learning, and external validation may be useful in future pandemics for appropriate resource allocation.

## Conclusions

In our retrospective cohort analysis of 7,429 COVID-19 positive patients at two community hospitals in Los Angeles, we found that age ≥ 60 years, triage oxygenation <90%, chronic kidney disease, and elevated BMI ≥30 kg/m^2 were among the risk factors most strongly associated with in-hospital mortality. Furthermore, our prediction tool using our triage parameters accurately predicted the outcome (death or survival) in 86% of the 1,429 patients in the internal validation testing cohort. This prediction tool may be useful to allocate resources and ration care during times of unprecedented surges.
